# Functional Profiling of In Vitro Reactivated Memory B Cells Following Natural SARS-CoV-2 Infection and Gam-COVID-Vac Vaccination

**DOI:** 10.3390/cells11131991

**Published:** 2022-06-21

**Authors:** Ekaterina A. Astakhova, Maria G. Byazrova, Gaukhar M. Yusubalieva, Sergey V. Kulemzin, Natalia A. Kruglova, Alexey G. Prilipov, Vladimir P. Baklaushev, Andrey A. Gorchakov, Alexander V. Taranin, Alexander V. Filatov

**Affiliations:** 1 Laboratory of Immunochemistry, National Research Center Institute of Immunology, Federal Medical Biological Agency of Russia, 115522 Moscow, Russia; ast_kat@mail.ru (E.A.A.); mbyazrova@list.ru (M.G.B.); a_prilipov@mail.ru (A.G.P.); 2 Department of Immunology, Faculty of Biology, Lomonosov Moscow State University, 119234 Moscow, Russia; 3 Department of Immunology, Institute of Medicine, Peoples’ Friendship University of Russia (RUDN University), 117198 Moscow, Russia; 4 Laboratory of Cell Technology, Federal Research and Clinical Center for Specialized Types of Medical Care and Medical Technologies of the FMBA of Russia, 115682 Moscow, Russia; gaukhar@gaukhar.org (G.M.Y.); serpoff@gmail.com (V.P.B.); 5 Laboratory of Immunogenetics, Institute of Molecular and Cellular Biology, Siberian Branch of the Russian Academy of Sciences, 630090 Novosibirsk, Russia; skulemzin@mcb.nsc.ru (S.V.K.); andrey.gorchakov@gmail.com (A.A.G.); taranin@mcb.nsc.ru (A.V.T.); 6 Laboratory of Gene Therapy of Socially Significant Diseases, Center for Precision Genome Editing and Genetic Technologies for Biomedicine, Institute of Gene Biology of the Russian Academy of Sciences, 119334 Moscow, Russia; natalya.a.kruglova@yandex.ru; 7 Laboratory of Molecular Genetics, N.F. Gamaleya National Research Center for Epidemiology and Microbiology, Ministry of Health of the Russian Federation, 123098 Moscow, Russia

**Keywords:** SARS-CoV-2, COVID-19, Gam-COVID-Vac vaccine, Sputnik V vaccine, memory B cells, humoral immunity, neutralizing antibodies

## Abstract

Both SARS-CoV-2 infection and vaccination have previously been demonstrated to elicit robust, yet somewhat limited immunity against the evolving variants of SARS-CoV-2. Nevertheless, reports performing side-by-side comparison of immune responses following infection vs. vaccination have been relatively scarce. The aim of this study was to compare B-cell response to adenovirus-vectored vaccination in SARS-CoV-2-naive individuals with that observed in the COVID-19 convalescent patients six months after the first encounter with the viral antigens. We set out to use a single analytical platform and performed comprehensive analysis of serum levels of receptor binding domain (RBD)-specific and virus-neutralizing antibodies, frequencies of RBD-binding circulating memory B cells (MBCs), MBC-derived antibody-secreting cells, as well as RBD-specific and virus-neutralizing activity of MBC-derived antibodies after Gam-COVID-Vac (Sputnik V) vaccination and/or natural SARS-CoV-2 infection. Overall, natural immunity was superior to Gam-COVID-Vac vaccination. The levels of neutralizing MBC-derived antibodies in the convalescent patients turned out to be significantly higher than those found following vaccination. Our results suggest that after six months, SARS-CoV-2-specific MBC immunity is more robust in COVID-19 convalescent patients than in Gam-COVID-Vac recipients. Collectively, our data unambiguously indicate that natural immunity outperforms Gam-COVID-Vac-induced immunity six months following recovery/vaccination, which should inform healthcare and vaccination decisions.

## 1. Introduction

Accurately forecasting the dynamics of COVID-19 spread requires a thorough understanding of the duration and breadth of immunity to SARS-CoV-2 antigens. According to the current estimates, one-third of the human population has already had COVID-19, and more than half has been fully vaccinated. Population immunity is a composite of infection-induced and vaccine-induced immunity. Both types of anti-SARS-CoV-2 immunity have been established to be at least partially protective. Natural immunity has been shown to reduce the risk of reinfection and COVID-19-associated morbidity and mortality, and lasts for 6–12 months [1]. Similarly, vaccination significantly lowers transmission and reduces disease severity, as well as disability and death risk. The efficacy of vaccination has been well documented in both short-term randomized controlled trials and over longer timescales [2,3]. Nevertheless, it remains actively debated which of the two types of SARS-CoV-2-specific immunity is longer-lived and more protective [4]. To address this important question, several population-based studies have been performed, where infection-induced and vaccine-induced immunities were compared.

Specifically, data from several large-scale outcome studies measuring the reinfection rate among COVID-19-recovered subjects and the breakthrough rate in vaccinated COVID-19-naïve individuals have been summarized [5]. This analysis led the authors to conclude that natural immunity in the convalescent subjects was superior in terms of protection than vaccination in the naïve individuals. Similar results have been reported in a retrospective observational study, where SARS-CoV-2-naïve vaccinees displayed higher risk for breakthrough infection than the previously infected individuals [6]. It is therefore quite reasonable to conclude that natural immunity may offer equal or greater protection against SARS-CoV-2 infection compared to vaccination [7]. 

Careful analysis of memory B-cell immunity elicited by natural infection and/or various vaccination/boosting regimens is an area of active ongoing research [4,5,6,7]. This is particularly important for lower-income countries and places where SARS-CoV-2 vaccination is no longer mandatory, properly controlled, or available, as immunity induced by the natural exposure to SARS-CoV-2 will likely remain the only factor limiting the spread of infection in such areas of the world.

In order to gain a deeper understanding of the immunological underpinnings underlying the differences between natural and vaccine-induced immunity, epidemiological studies should be complemented with serological and cellular laboratory-based studies. SARS-CoV-2 infection and vaccination result in similar and robust immunity and immunological memory [8,9], which is manifested by production of serum virus-neutralizing antibodies, as well as by formation of T- and B-lymphocyte memory cells. In both immunization routes, humoral and cellular immunity against SARS-CoV-2 have been reported to persist for up to 11 months [10].

Whereas extensive studies focusing on the immunity induced by either natural exposure or vaccination are available, very few head-to-head comparisons based on a single analytical platform have been performed to date. Intriguingly, the conclusions reached appear to be conflicting. Specifically, mRNA vaccination has been reported to result in higher antibody titers featuring broader neutralization potency compared to the levels observed upon natural infection [11,12]. Further, mRNA- and adenovirus-based SARS-CoV-2 vaccines provided higher seroconversion rates [13]. In contrast, other studies argued that natural immunity provides greater magnitude of effector T-cell responses [14], and more mature memory B-cells (MBCs) [15]. Typically, assessment of humoral immunity against SARS-CoV-2 has involved measurements of anti-S and anti-RBD antibody levels, as well as studies of the functional properties of circulating antibodies in virus-neutralization assays [16]. In contrast, evaluation of B-cell immunity has been largely limited to enumeration of S- and RBD-specific MBCs regardless of the functional quality of antibodies that are secreted by the reactivated MBCs [17]. 

Gam-COVID-Vac (Sputnik V) is a COVID-19 adenovirus-based two-part vaccine. Several clinical trials (~20 trials currently ongoing) have demonstrated its excellent safety and efficacy profiles [18,19,20], which has led to the authorization of Gam-COVID-Vac in nearly 70 countries [21]. Earlier, features of MBC responses induced by vaccination with Gam-COVID-Vac soon after the first vaccine dose were explored [22]. In the present study, we aimed to analyze and compare B-cell responses following natural SARS-CoV-2 infection and Gam-COVID-Vac vaccination six months after the first encounter with the viral antigens. Our study relies on four methods: flow cytometry of RBD-binding circulating cells, ELISpot measurements of SARS-CoV-2-specific antibody-secreting cell (ASC) frequencies, ELISA-based quantification of the overall level of MBC-derived antibodies, and virus neutralization activity of MBC-derived antibodies. This methodological combination represents a more comprehensive set of approaches to assess and compare side-by-side MBC responses and MBC functionality in the contexts of natural and/or vaccine-induced anti-SARS-CoV-2 immunity.

## 2. Materials and Methods

### 2.1. Study Volunteers

A cohort of Gam-COVID-Vac recipients and COVID-19 convalescent patients was enrolled in December 2020 at the National Research Center Institute of Immunology of the Federal Medical Biological Agency of Russia. None of the participants were pregnant, immunodeficient, or receiving immunosuppressive treatment. Vaccine recipients were immunized by two doses of Gam-COVID-Vac with a 21-day interval between the doses. Written informed consent was obtained from each of the study participants before performing any study procedures. The study protocol was reviewed and approved by the Medical Ethical Committee of the Institute of Immunology (#12-1, 29 December 2020). The study was conducted in accordance with the Declaration of Helsinki and the Guidelines for Good Clinical Practice.

### 2.2. Blood Sample Collection and Processing

Whole-blood samples were collected into heparinized vacutainer tubes (Sarstedt, Nümbrecht, Germany). Peripheral blood mononuclear cells (PBMCs) were isolated via density gradient centrifugation. Plasma samples were stored at −70 °C. B cells were purified from freshly isolated PBMCs by negative selection using the Dynabeads Untouched human B-cells kit (Thermo Fisher Scientific, Waltham, MA, USA) and were used in the assays immediately. As a pre-pandemic control, we used blood samples cryopreserved no later than September 2019.

### 2.3. ELISA Quantification of RBD-Specific IgG, IgA, and IgM Antibodies 

The levels of RBD-specific antibodies were determined using an in-house RBD IgG/IgA/IgM quantitative ELISA test [22]. Human monoclonal antibody iB12 [23] was used as the calibrator for quantitative ELISA test. Test results were expressed as absolute (ng/mL) or relative units (RU).

### 2.4. Flow Cytometry

Total MBCs were detected after staining for 30 min with the following antibody cocktail containing CD19-Alexa Fluor^®^ 488 (clone LT19), CD27-PECy5.5 (clone LT27), (all of which were produced in-house earlier), and IgD-APC-Cy7 (clone IA6-2, Sony Biotechnology, San Jose, CA, USA). Antigen-specific MBCs were detected by double staining with fluorescently labeled recombinant RBD protein from the WT virus. RBD was fluorescently labeled with phycoerythrin (RBD-PE) or allophycocyanin (RBD-APC). Production of recombinant RBD protein subunit from SARS-CoV-2 (isolate Wuhan-Hu-1) conjugated to PE or APC was described earlier [22,24]. As a negative control, we used the samples stained with an irrelevant PE-labeled protein Bet v 1, which is the major birch allergen. Following surface staining, cells were washed twice with PBS and analyzed on a CytoFLEX S flow cytometer (Beckman Coulter, Krefeld, Germany). For each specimen, at least 800,000 single CD19^+^ events were recorded. Data analysis was performed using FlowJo Software (version 10.6.1, Tree Star, Ashland, OR, USA).

### 2.5. B-Cell Stimulation and ELISpot Assay

Quantification of SARS-CoV-2-specific IgG ASCs was performed by enzyme-linked immunosorbent spot (ELISpot) assay, as described previously [24]. Briefly, purified B-cells were stimulated for 7 days at a density of 5 × 10^3^ B cells/well in 96-well plates in the presence of 25 ng/mL interleukin-21 (IL-21; PeproTech, Cranbury, NJ, USA) and mitomycin-treated feeder A549 cells stably expressing CD40L (A549-CD40L, 1 × 10^5^ cells/well) in DMEM medium supplemented with 10% fetal bovine serum, 24 µg/mL of gentamicin, 10 mM l-glutamine, 1 mM sodium pyruvate, and 10 mM HEPES (all Paneko, Moscow, Russia). These growth conditions provided appropriate MBC expansion and differentiation into MBC-derived ASCs. The supernatants from IL-21/CD40L activated B-cells containing MBC-derived antibodies were collected and used for measuring the levels of antibody binding to SARS-CoV-2 RBD in ELISA or in pseudotyped virus neutralization assays. Multiscreen 96-well Filter Plates with polyvinylidene difluoride membrane (Merck Millipore, Cork, Ireland) were coated with 10 mg/mL of recombinant RBD. IL-21/CD40L stimulated B-cells were plated at a density of 100–1000 or 10,000–30,000 of cells per well in duplicate for determination of total and RBD-specific IgG ASCs, respectively. After incubation for 16 h at 37 °C, 5% CO_2_, the cells were thoroughly removed with a washing buffer. Spots were developed by incubating with human IgG-specific biotinylated rabbit antibodies (R&D Systems, Minneapolis, MN, USA) at a 1:100 dilution followed by five sequential washes and adding streptavidin alkaline phosphatase conjugate (R&D Systems) and Substrate Reagent from B Cell ELISpot Development Module (R&D Systems). ELISpot images were acquired using a CTL ImmunoSpot^®^ analyzer (CTL, New York, NY, USA). Spots were counted using CTL’s ImmunoSpot^®^ software. Wells coated with an irrelevant protein Bet v 1 served as negative controls.

### 2.6. Pseudotyped Virus Neutralization Assay (pVNA)

To produce SARS-CoV-2 Spike-pseudotyped lentiviral particles, we used 3 plasmids: HIV-1 packaging pCMVΔ8.2R (Addgene, Watertown, MA, USA), transfer pUCHR-GFP (Addgene), and envelope pCAGGS-Swt-Δ19 [22]. The latter plasmid encodes a codon-optimized ancestral Wuhan-Hu-1 Spike (Δ19) protein lacking 19 C-terminal amino acid residues. Based on pCAGGS-Swt-Δ19, substitutions found in the Delta variant Spike (T19R, G142D, 156del, 157del, R158G, L452R, T478K, D614G, P681R, D950N) were introduced into the Spike coding region resulting in a pCAGGS-SDelta-Δ19 plasmid. Omicron (BA.1) Spike-encoding CDS was gene synthesized (Genewiz, South Plainfield, NJ, USA) and cloned into the pCAGGS-Swt-Δ19 plasmid to obtain pCAGGS-SOmi-Δ19. Omicron Spike had the following substitutions relative to the ancestral Wuhan-Hu-1 sequence: A67V, Δ69-70, T95I, G142D, Δ143-145, Δ211, L212I, ins214EPE, G339D, S371L, S373P, S375F, K417N, N440K, G446S, S477N, T478K, E484A, Q493R, G496S, Q498R, N501Y, Y505H, T547K, D614G, H655Y, N679K, P681H, N764K, D796Y, N856K, Q954H, N969K, L981F.

One day before transfection, 3.6 × 10^6^ HEK293T cells were seeded onto 100 mm Petri dishes. The next day, 2.7 µg of pCAGGS-S (wt, Delta, or Omicron)-Δ19, 8.7 µg of pCMVΔ8.2R, and 13.2 µg of pUCHR-GFP were mixed together and transfected into HEK293T cells using calcium phosphate-based protocol. Then, 48 h later, supernatant from transfected HEK293T cells was harvested and virus-like particles (VLPs) were concentrated by centrifugation at 30,000× *g* for 2.5 h. Aliquots of concentrated VLPs were frozen at −70 °C. A standard dose of VLPs that provided infection of 50% of target cells (HEK293T cells stably expressing human ACE2) was used across all experiments. For pVNA, 20 µL of serially diluted serum samples or supernatants from cultures of IL-21/CD40L-stimulated B-cells was pre-mixed with 10 µL of VLPs, incubated for 1 h and added to HEK293T-ACE2 cells plated at 5 × 10^3^ cells/well in 10 µL of medium in 96-well plates. Cells were then cultured for 48 h. Each assay was performed in duplicate. After that, cells were resuspended and the percentage of GFP^+^ cells was enumerated using a CytoFLEX S flow cytometer (Beckman Coulter, Krefeld, Germany). Neutralization half-maximal inhibitory plasma dilution (ID_50_) values were determined using normalized nonlinear regression with Sigmoidal, 5PL (GraphPad Software, San Diego, CA, USA).

### 2.7. Statistical Analysis

The Kruskal–Wallis H test was used for comparison between multiple groups or Friedman test for pairwise comparison. *p* < 0.05 was considered statistically significant. Nonparametric Spearman correlations analyses were used to determine associations between the analyzed parameters. All statistical analyses were carried out using GraphPad Prism version 8.4.3 (GraphPad Software, San Diego, CA, USA). Numbers of replicates and experiments for each dataset are indicated in the figure legends. Data are presented as median values and interquartile ranges (IQR). Asterisks indicate significant difference between the groups, * *p* < 0.05, ** *p* < 0.01, *** *p* < 0.001, **** *p* < 0.0001, ns = not significant.

## 3. Results

### 3.1. Study Design

Multiple lines of evidence indicate that the duration, magnitude, and breadth of immune responses in vaccinees is heavily dependent on whether they were previously infected with SARS-CoV-2 or not [9,25,26]. In order to identify the study participants who were infected prior to vaccination, all the serum samples were tested for the presence of anti-nucleocapsid (N) antibodies, as N-encoding sequences are absent from the Gam-COVID-Vac vaccine. As a rule, presence of the anti-N antibodies in the sera was associated with self-reported mild COVID-19 symptoms. Based on the anti-N antibody testing and vaccination status, study participants were stratified into three groups: (i) SARS-CoV-2-naïve individuals, who received Gam-COVID-Vac (N/V; *n* = 15); (ii) previously SARS-CoV-2-infected Gam-COVID-Vac-vaccinated individuals (PI/V; *n* = 13); (iii) previously SARS-CoV-2-infected unvaccinated (PI/unV; *n* = 26) individuals. All PI/V and PI/unV subjects had a PCR-confirmed SARS-CoV-2 infection. All PI/V (*n* = 13) and some of the PI/unV group (*n* = 11) participants had mild COVID-19 without hospitalization, moderate COVID-19 requiring hospitalization (*n* = 13) or severe COVID-19 with intensive care unit (ICU) admission (*n* = 2). All the vaccinated participants received two doses of Gam-COVID-Vac vaccine in the period between January and April 2021. The above study groups were overall similar in terms of the male/female ratio and displayed very minor age disparities (median age 60, 42, and 51 for N/V, PI/V, and PI/unV groups, respectively; *p* values ≥ 0.24) (Table 1).

On average, peripheral blood samples were collected on day 180 (median, range 166–208) after the event, which was either the first dose of Gam-COVID-Vac in the N/V and PI/V groups, or full recovery in the PI/unV group. It must be noted that all the previously infected participants reported experiencing COVID-19 symptoms between May 2020 and February 2021, when B.1 and B.1.1 were the dominant circulating lineages in the Moscow region [19,27,28]. Consequently, both the vaccinated and the previously infected study participants received a homologous rather than a heterologous vaccine encoding the ancestral Spike, which was nearly identical to the Spike in the circulating viral lineages at that time. The overall study design is illustrated in Figure S1.

### 3.2. Serum Antibody Responses to SARS-CoV-2 Antigens from COVID-19 Convalescent Patients and Gam-COVID-Vac Vaccinees

**Figure 1:**
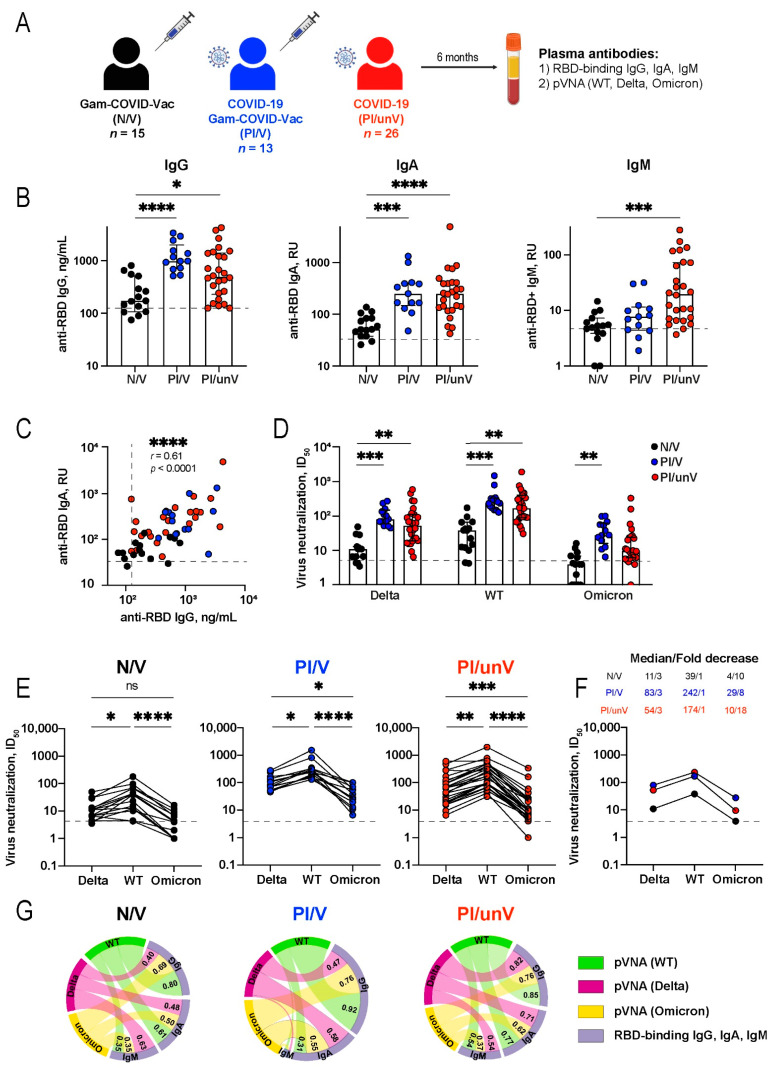
RBD-binding and virus-neutralizing activity of sera from COVID-19 convalescent patients and Gam-COVID-Vac recipients. (**A**) Study design. (**B**) Serum IgG (left panel), IgA (middle panel) or IgM (right panel) antibody binding to SARS-CoV-2 RBD, measured by ELISA. IgA and IgM levels are shown as relative units (RU) against a standard convalescent serum. Each dot represents a concentration or RU for each serum sample. The dotted lines indicate the threshold for positivity (anti-RBD IgG = 126 ng/mL, IgA = 33 RU, IgM = 4.0 RU). (**C**) Spearman’s correlation between the serum levels of anti-RBD IgG and IgA. (**D**) Virus-neutralizing activity (ID_50_) of serum samples against lentiviral particles pseudotyped with Spike protein from WT SARS-CoV-2, as well as Delta and Omicron variants. (**E**) Analysis of serum neutralization activities (ID_50_) against WT-, Delta-, and Omicron-pseudotyped lentiviral particles. Lines connect ID_50_ values from the same individual. (**F**) Summary of virus-neutralizing activities of serum samples against the WT, Delta, and Omicron Spike-pseudotyped lentiviral particles. (**G**) Chord diagram illustrating the Spearman’s correlations between virus neutralization (ID_50_) of WT, Delta and Omicron variants and the levels of anti-RBD IgG, IgA, and IgM. Chord widths are proportional to the correlation coefficients, whose numerical values are indicated on the corresponding chords. Black, blue, and red symbols indicate naïve vaccinated (N/V, *n* = 15), previously SARS-CoV-2 infected Gam-COVID-Vac vaccinated (PI/V; *n* = 13), and previously SARS-CoV-2 infected unvaccinated (PI/unV; *n* = 26) individuals. Symbols connected by solid lines (**E**) represent virus-neutralizing activities considered for each individual. All the experiments were performed at least in triplicate. Data are presented as median ± IQR. The dotted lines indicate the threshold for positivity. Statistics were calculated using the Kruskal–Wallis test (**B**,**D**), or the Friedman test for pairwise comparison (**E**,**F**). * *p* < 0.05, ** *p* < 0.01, *** *p* < 0.001, **** *p* < 0.0001, ns, not significant. ID_50_, half-maximal inhibitory dilution; IQR, interquartile range; RBD, receptor-binding domain; RU, relative units.

Although studying the details of humoral immunity was not the focus of our analysis, in order to comprehensively describe B-cell immunity following SARS-CoV-2 infection or vaccination, we first compared major antibody responses in the sera from COVID-19 convalescent patients and Gam-COVID-Vac vaccinees (Figure 1A). Namely, the presence of RBD-specific IgG, IgA, and IgM was evaluated by ELISA. Six months following infection or vaccination, vast majority of study participants remained seropositive (Figure 1B). Notably, the levels of serum RBD-specific IgGs in the PI/V (median = 985 ng/mL) and PI/unV (median = 498 ng/mL) groups were significantly higher than those in the N/V recipients (median = 173 ng/mL; *p* < 0.0001 and *p* = 0.0229, respectively). No significant differences were observed between the PI/V and PI/unV individuals.

Relative levels of RBD-specific IgAs in the three studied groups followed the same trend as for IgGs, with even more pronounced differences between the naïve and previously infected individuals (median for N/V = 51 RU; N/V vs. PI/V *p* = 0.0002; N/V vs. PI/unV *p* < 0.0001) (Figure 1B, middle panel). As for RBD-specific IgM levels, noticeable differences were only observed when comparing N/V vs. PI/unV individuals (*p* = 0.0003) (Figure 1B, right panel). Predictably, magnitudes of IgG and IgA responses in individual study participants were well correlated (Spearman’s r = 0.61, *p* < 0.0001) (Figure 1C).

Next, in order to assess the functional quality of circulating antibodies, virus neutralization experiments were performed. To this end, we used a neutralization assay with lentiviral particles pseudotyped with wild-type as well as Delta and Omicron (BA.1) variant SARS-CoV-2 Spike proteins. Plasma virus-neutralizing activity against WT pseudovirus was detectable across all PI/V (*n* = 13) and PI/unV (*n* = 26) group individuals, as well as most (86.7%, 13/15) of the SARS-CoV-2 naive subjects (Figure 1D). In the previously infected cohort, a significantly higher ID_50_ was observed both in vaccinated (median value 242, IQR 164–340) and unvaccinated subjects (median value 175, IQR 91–416) compared to the naïve vaccinees (median value 39, IQR 12–67; N/V vs. PI/V *p* = 0.0009; N/V vs. PI/unV *p* = 0.0017).

Plasma neutralization potency decreased when samples were tested against Delta pseudoviral particles (Figure 1E,F). In all groups, ID_50_ values against Delta Spike were, on average, three-fold lower compared to the WT (*p* = 0.0185, *p* = 0.0324, and *p* = 0.0026 for N/V, PI/V and PI/unV groups, respectively). The plasma neutralization capacity dropped even further when Omicron Spike pseudoviruses were used. ID_50_ values for Omicron Spike were, on average, 10-, 8-, and 18-fold lower compared to the WT (*p* < 0.0001, *p* < 0.0001, and *p* < 0.0001 for N/V, PI/V and PI/unV groups, respectively).

Various degrees of correlation were observed between the virus-neutralizing activities of plasma samples against WT Spike-pseudotyped lentiviral particles and the RBD-specific IgG, IgA, and IgM responses (Figure 1G). pVNA-derived ID_50_ values for WT Spike and IgG responses in PI/V and PI/unV individuals (Spearman’s r = 0.92 and 0.85, respectively) displayed the strongest correlation. No or low correlation was found for pVNA ID_50_ and IgA or IgM (Figure 1G).

### 3.3. Memory B-Cell Response

Antibodies present in the serum are known to be produced by plasma cells found in the bone marrow, lymph nodes, and spleen, and also by B1 cells and plasmablasts. Upon pathogen/antigen re-encounter, most of the antibody response is derived from the activated memory B-cells. Therefore, serum antibody levels are only part of the humoral SARS-CoV-2-specific immunity, and the dynamics of RBD-specific MBCs plays a prominent role. In our work, MBCs were enumerated using two complementary approaches: by their ability to bind fluorescently labeled RBD and their ability to secrete anti-RBD antibodies in the ELISpot assay.

**Figure 2:**
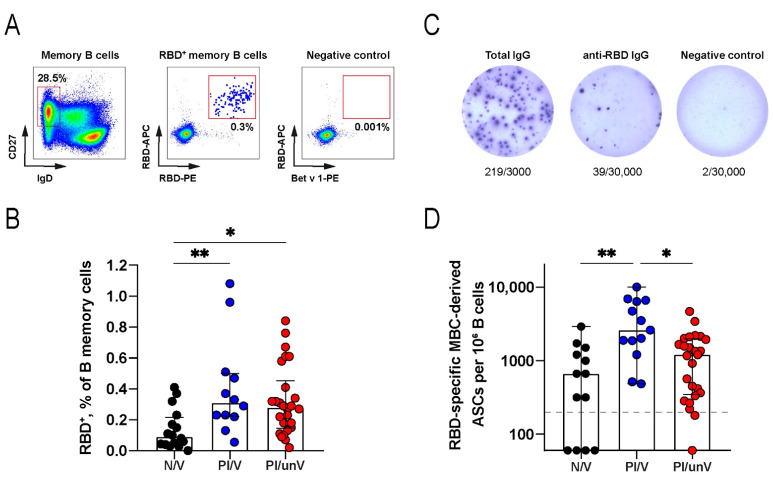
Analysis of circulating memory B-cells in COVID-19 convalescent patients and Gam-COVID-Vac recipients. (**A**) Representative flow cytometry dot plots showing dual PE–RBD- and APC–RBD-binding MBCs. Numbers inside the plots indicate the percentage of events specific to the respective gates. (**B**) RBD^+^ MBCs as a percentage of all memory B-cells (CD19^+^CD27^+^IgD^−^). (**C**) Representative ELISpot showing SARS-CoV-2-specific MBC-derived ASCs. Purified B-cells were stimulated with IL-21/CD40L for 7 days and then incubated in ELISpot plates for 16 h to detect ASCs secreting total (left), RBD-specific (middle) or IgG against irrelevant Bet v 1 protein (right). The numbers indicated below the wells represent positive dots and the total number of cells in the well. (**D**) RBD-specific MBC-derived ASCs per 10^6^ B-cells from blood samples of naïve vaccinated (N/V, *n* = 15), previously SARS-CoV-2-infected Gam-COVID-Vac-vaccinated (PI/V; *n* = 13), and previously SARS-CoV-2-infected unvaccinated (PI/unV; *n* = 26) individuals. * *p* < 0.05, ** *p* < 0.01.

Total MBCs were identified by flow cytometry as having a CD19^+^CD27^+^IgD^−^ surface phenotype (Figure 2A). Given that RBD^+^ MBCs are relatively rare, to minimize the contribution of false-positive events, cells were dual labeled with phycoerythrin- and allophycocyanin-conjugated RBD (RBD-PE and RBD-APC). As a negative control, cells were stained with PE and APC conjugates of an irrelevant Bet v 1 protein. The frequency of Bet v 1^+^ in our samples was below 0.001%, which was taken as a cutoff threshold for background binding.

As reported previously by our group and others, RBD^+^ MBCs are formed both after vaccination and following COVID-19 [9,24,29]. Here, we aimed to quantify RBD^+^ MBCs in either cohort six months following vaccination and convalescence, respectively. In PI/V and PI/unV subjects, comparable frequencies of RBD^+^ MBCs were observed (median percentage of RBD^+^ cells in total MBC population: 0.31% and 0.28% in PI/V and PI/unV groups, respectively) (Figure 2B), and were well above the Bet v 1-derived cutoff (0.001%). These numbers were approximately three-fold higher than in SARS-CoV-2-naive individuals (median 0.09; N/V vs. PI/V *p* = 0.0075; N/V vs. PI/unV *p* = 0.0123) (Figure 2B). Thus, taking a snapshot of 6 months into consideration, previously infected subjects outperformed naïve vaccinees in terms of RBD^+^ MBC counts. 

In resting state, MBCs have surface-expressed antibodies, but generally do not secrete them. We asked whether MBCs from our cohorts were functionally active and secreted RBD-specific antibodies upon polyclonal CD40L/IL-21 stimulation. Using ELISpot assay, this was indeed confirmed (representative images are shown in Figure 2C). As negative/background controls, pre-pandemic blood samples as well as ELISpot using irrelevant Bet v 1 protein were used. These important controls helped establish a cutoff for positivity at about 150 spots per million B-cells.

Six months following the event, RBD-specific ASCs above the baseline were detected in 60% (9/15), 100% (13/13), and 96% (25/26) of the subjects from N/V, PI/V, and PI/unV groups, respectively (Figure 2D). Statistically significant differences were only observed for N/V and PI/V groups (*p* = 0.001) and PI/V and PI/unV groups (*p* = 0.018). Although RBD-specific ASC numbers were the highest in the PI/V participants, the median value for this group was well below the numbers previously reported for the samples collected during the acute phase of SARS-CoV-2 infection or 85 days following vaccination [22,24]. We did not observe any correlation between the numbers of RBD-binding cells and RBD-specific ASCs. Perhaps this is due to the fact that these B-cell subsets represent different stages of antigen-specific MBCs maturation.

### 3.4. The Functionality of MBC-Derived SARS-CoV-2 Antigen-Specific Antibodies

While both RBD-binding and ELISpot assays are instrumental in assessing the frequencies of circulating antigen-specific MBCs, they do not inform on the overall antibody secretion levels, nor do they help understand whether the antibodies produced by activated antigen-specific MBCs have any virus-neutralizing activity. To address these important questions, purified B-cells were activated in vitro for 7 days in the presence of CD40L and IL-21 driving MBC expansion and differentiation into MBC-derived ASCs. ASCs obtained this way were used in ELISpot assay, as described above, and activities of RBD-binding and virus-neutralizing antibodies in the supernatants were measured.

**Figure 3:**
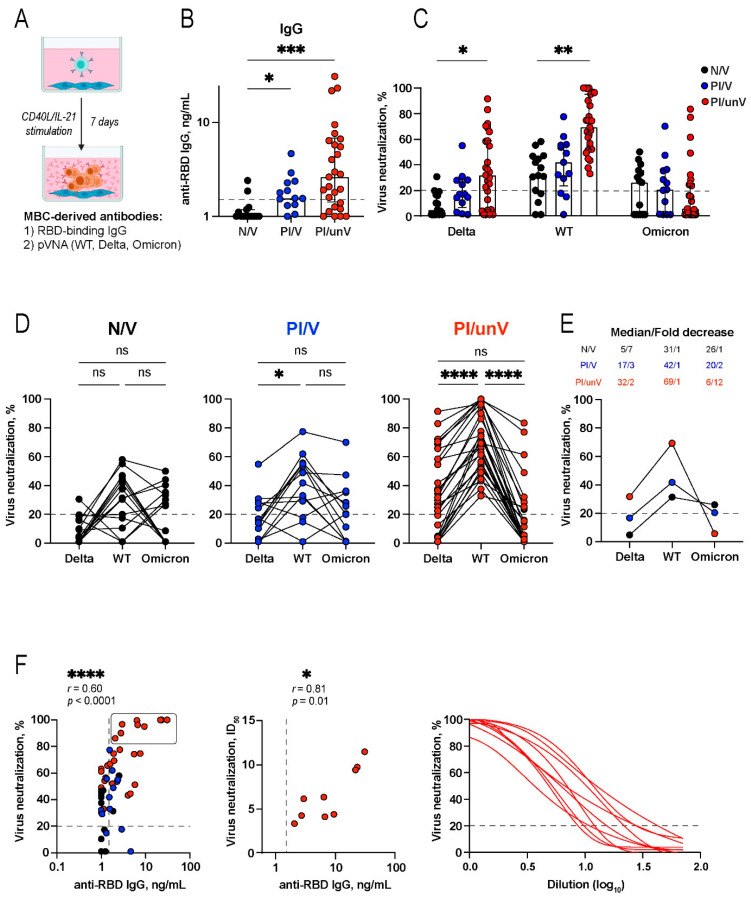
Analysis of MBC-derived antibody response of in vitro reactivated memory B-cells elicited by natural SARS-CoV-2 infection and by Gam-COVID-Vac vaccination. (**A**) Schematic representation of the MBC-derived antibody assay. (**B**) Production of RBD-specific IgG in cultures of IL-21/CD40L-stimulated B-cells evaluated using ELISA. (**C**) Virus-neutralizing activity of MBC-derived antibodies against WT strain and Delta and Omicron variants in different study groups. Infected (% GFP^+^) cells relative to no antibody controls. (**D**) Comparison of neutralization activity of individual supernatant samples against WT-, Delta-, and Omicron-pseudotyped lentiviral particles. Symbols connected by solid lines represent virus-neutralizing activities considered for each individual. (**E**) Summary of virus-neutralizing activities of MBC-derived antibodies against WT strain and Delta and Omicron variants. (**F**) Spearman’s correlation between the levels of anti-RBD IgG and % of WT virus neutralization (left panel) or ID_50_ (middle panel). Nine most active supernatants on the left panel are marked with a rectangle. Nonlinear regression curves for virus neutralization of the nine most active supernatants (right panel). Statistics were calculated using the Kruskal–Wallis test (**B**,**C**), or the Friedman test for pairwise comparison (**D**,**E**). Data are from 3 (**B**) or 2 (**C**–**F**) independent experiments, each in duplicate wells, and the data are shown as median ± IQR. Black, blue, and red symbols indicate naïve vaccinated (N/V, *n* = 15), previously SARS-CoV-2-infected Gam-COVID-Vac-vaccinated (PI/V; *n* = 13), and previously SARS-CoV-2-infected unvaccinated (PI/unV; *n* = 26) individuals. * *p* < 0.05, ** *p* < 0.01, *** *p* < 0.001, **** *p* < 0.0001, ns, not significant.

First, we evaluated the presence of RBD-specific IgG, IgA, and IgM using ELISA (Figure 3B and Figure S2). B-cell samples collected before the COVID-19 pandemic were used as a negative control. A cutoff threshold twice above the level of RBD-specific reactivity in the pre-pandemic control was arbitrarily chosen. In some samples, pronounced secretion of RBD-specific MBC-derived antibodies was observed. This MBC-derived antibody response was most often registered in PI/unV individuals. In this group, 54% (14/26), 38% (10/26), and 61% (16/26) of study participants had RBD-specific IgG, IgA, and IgM levels above the pre-defined cutoff, respectively. In the N/V group, RBD-specific MBC-derived IgG levels were overall inferior to those found in other groups (N/V vs. PI/V, *p* = 0.0341; N/V vs. PI/unV, *p* = 0.0006). Only 13% (2/15), 38% (5/15) and none (0/15) of N/V participants displayed secreted RBD-specific IgG, IgM, and IgA at the levels above the cutoff, respectively.

Next, we proceeded to measure the virus-neutralizing activity of MBC-derived antibodies using human immunodeficiency virus-1 (HIV-1) pseudotyped with Spike from SARS-CoV-2 WT, Delta, and Omicron. Antibody concentration in the supernatants is generally 2–3 orders of magnitude lower than in the plasma, so undiluted samples were initially used for measurements. The cutoff value (20%) was established based on the results obtained with supernatants from historic samples. Figure 3C shows that the strongest virus-neutralizing activity against SARS-CoV-2 WT Spike-pseudotyped lentiviral particles was found in the supernatants from the PI/unV group (median value 69%, IQR 52–95), which is ~two times higher that of the virus neutralization observed in supernatants derived from N/V participants (median value 31%, IQR 17–47; *p* = 0.0049).

As expected, virus-neutralization potency was significantly lower for Delta variant compared to WT pseudovirus. Despite the overall decrease in neutralization, the potency of supernatants from PI/unV individuals (median value 32%, IQR 5–59) was still above the values obtained on N/V samples (median value 5%, IQR 1–16; *p* = 0.0145) (Figure 3C, middle panel). For the most part, nearly background virus-neutralizing activity of MBC-derived supernatants against Omicron-pseudotyped lentiviral particles was observed across all three groups of donors. Only 53% (8/15), 54% (7/13), and 35% (9/26) of individuals from N/V, PI/V, and PI/unV groups, respectively, displayed virus-neutralizing activity above the threshold (Figure 3C, right panel). Figure 3D,E summarize the values of virus-neutralizing activities against WT, Delta, and Omicron Spike-pseudotyped lentiviral particles in each of the groups of donors. PI/unV group had the highest neutralization levels. In this group, the percent of neutralization against Delta and Omicron was reduced by a median of 2- and 12-fold (*p* < 0.0001 and *p* < 0.0001), respectively, compared to WT (Figure 3D,E).

Notably, the levels of RBD-specific MBC-derived IgGs were moderately correlated with the virus-neutralizing potency of supernatants (Spearman’s r = 0.60, *p* < 0.0001; Figure 3F, left panel). It is worth noting that the top nine supernatants could provide 80-100% neutralization of SARS-CoV-2 WT Spike pseudovirus. Clearly, neutralizing antibodies in these samples were present at saturating levels. In order to measure neutralization potency more accurately, additional experiments using serially diluted samples were performed and ID_50_ values for these samples were obtained (Figure 3F, right panel). This translated to the increase in Spearman’s correlation between RBD-specific MBC-derived IgG levels and the virus-neutralizing capacity of supernatants (r = 0.81, *p* = 0.02) (Figure 3F, middle panel).

## 4. Discussion

Immunity elicited by SARS-CoV-2 infection and vaccination-induced immunity share multiple overlapping features, yet there are also important differences in terms of how the human body will respond to the pathogen upon re-encounter later in life. In both cases, SARS-CoV-2 Spike protein serves as the key target for neutralizing antibodies. However, the two routes of immunization differ significantly in the antigen dosage and stability, exposure site, type, and magnitude of accompanying inflammatory processes—leaving aside the multitude of non-Spike-directed immune responses. Cumulatively, these factors may translate into quantitative and qualitative differences in the elicited protective immunity. Our study is geared towards delineating and comparing the functional features of vaccine- and infection-induced MBCs. Notably, the vaccine used in our work, an adenovirus vector-based Gam-COVID-Vac, encodes SARS-CoV-2 Spike in its native, non-stabilized form, unlike in many other vaccines [3], which makes the comparisons more adequate.

We aimed to compare several immunological parameters in infection-free vaccinated individuals and in COVID-19-convalescent subjects six months after vaccination/recovery. These included anti-RBD and virus-neutralizing activity of serum antibodies, frequency of RBD-specific circulating MBCs, frequency of MBC-derived ASCs, as well as RBD-specific and virus-neutralizing activity of MBC-derived antibodies. Our data indicate that immunity acquired during natural SARS-CoV-2 infection outperforms Gam-COVID-Vac-induced immunity in each of these parameters.

One of the first manifestations of the unfolding immune response is appearance of a transient population of plasmablasts. Accordingly, the earliest differences between natural and vaccine-induced immunity become detectable at the plasmablast stage. The acute phase of COVID-19 is known to be accompanied with massive plasmablast expansion [30], with most of these cells formed via an extra-follicular B-cell activation pathway [31]. Unlike during infection, Gam-COVID-Vac vaccination results in a moderate expansion of plasmablasts [22].

Comparison of humoral responses elicited by infection and vaccination is not as straightforward. First and foremost, these responses differ significantly in their dynamics. Whereas COVID-19 is accompanied with rapid and sharp production of Spike-specific and virus-neutralizing antibodies, vaccination leads to a more durable and robust antibody response [32]. Interestingly, mRNA-based and vectored vaccines have been shown to provide higher seroconversion rates than are observed in the convalescents [13].

Regarding the levels and specificity of antibodies, the existing body of evidence is somewhat mixed. Antibody levels have been reported to be higher in vaccinated individuals than in COVID-19 recovered patients [11,12,13]. Other studies confirm these findings, but only when applied to mildly or moderately ill rather than to severely ill patients [33]. On the one hand, mRNA vaccine-induced antibodies display a broader reactivity spectrum than those induced by natural infection [11]. On the other hand, the specificity of mRNA vaccine-induced antibodies appears limited to RBD, whereas the antibodies elicited by infection are less RBD-centered [34]. Infection- and vaccine-induced antibodies are also functionally distinct, with the former being largely virus-neutralizing and the latter being merely Spike-binding [16].

Our data on the serum antibodies are in contrast with the published reports exploring the post-infection and post-vaccination immunity in the context of SARS-CoV-2. These discrepancies may be attributable to the vaccines being compared, differences in sampling time, and disease severity in COVID-19 patients. Most of such studies have involved the samples from mRNA vaccine recipients, whereas our data are based on the adenovirus vector vaccine Gam-COVID-Vac, which has so far received less attention. Serum antibodies, although important, are clearly not the only nor the major predictors of long-term protection against SARS-CoV-2 infection. 

The T-cell arm of the adaptive immune response also makes a significant contribution to general immunity. Both SARS-CoV-2 infection and vaccination are known to elicit T-cell responses, with the former providing stronger T-cell reactivity [14]. Following antigen exposure, the titers of neutralizing antibodies progressively decline with time, and long-term protection against reinfection becomes largely controlled by MBCs. Following recovery, antibody sequences in these cells undergo affinity maturation, so that more potent virus-neutralizing antibodies are secreted upon antigen re-encounter [35].

Previous studies have shown that individuals who were previously infected with SARS-CoV-2 display more robust and broader immune responses to vaccination compared to naïve individuals [9,29,36]. However, the differences between these groups are not restricted to the magnitude and breadth of the response, as different proportions of classic, plasmablast-like, and atypical MBC subsets are formed [15]. Compared to Gam-COVID-Vac vaccination, natural SARS-CoV-2 infection leads to higher frequencies and longer maintenance of RBD^+^ MBCs. Our conclusions apply to the individuals who received two doses of Gam-COVID-Vac vaccine. It cannot be excluded, however, that after multiple vaccinations, the interplay between the immunity elicited by SARS-CoV-2 infection and that induced by the vaccination will be different.

Many MBC-focused studies are limited to MBC quantification using flow cytometry and B-cell ELISpot assay [17]. The mere presence of MBCs, however, does not equate function, and exploring the functional properties of MBC-derived antibodies, as demonstrated in our work, therefore appears highly warranted. MBC functionality can be analyzed at the clonal level by tracing the levels of antibody substitutions, as well as by establishing the breadth of neutralization of recombinant monoclonal antibodies. Clonal approaches indeed provide a very detailed image of the maturation process, but typically suffer from very low throughput.

In this study, we monitored the MBC functionality at the polyclonal level using an in vitro reactivation test. The levels of neutralizing MBC-derived antibodies in vaccinated-only individuals were found to be significantly lower than those following natural infection. Our data are in excellent agreement with the recent report that SARS-CoV-2-induced MBCs appear to be more important during the secondary response compared to the vaccine-induced MBCs [15]. In order to try to predict whether the acquired immunity is going to be protective in the face of emerging viral variants, the activity of serum antibodies is typically tested in virus-neutralization assays. Given the well-established role of MBC antibody responses upon antigen re-encounter later in life, it appears advisable to include this frequently overlooked cellular compartment in analyses, which would help prospectively assess the risks of reinfection/breakthrough more accurately. Humoral immunity is frequently characterized by the term “seroprevalence”. Likewise, MBC responses are probably best described by MBC-prevalence, which reflects the proportion of individuals who developed virus-neutralizing antibodies after MBC reactivation. Higher MBC-prevalence in the naïve subgroup is a clear evidence of ongoing MBC maturation leading to antibodies with higher affinity and avidity. 

Obviously, individual variation is always a confounding factor in population studies, and both high and low responders were present in the naïve group of study participants. In all likelihood, the convalescent group was even more heterogeneous, as individual differences were overlaid with differences in the viral dose, duration of viremia, and disease severity. These factors are inherently difficult to take into account when aiming to adequately compare SARS-CoV-2 natural immunity to vaccine-induced immunity. Another clear limitation of our study is that the sample sizes were low, which has prevented us from establishing the possible advantages of hybrid immunity formed upon vaccination of COVID-19 convalescent subjects.

## 5. Conclusions

We demonstrated that upon re-stimulation, MBCs from convalescent individuals secrete more potent antibodies compared to the MBCs from SARS-CoV-2-naïve vaccinees. Taken together, our observations further support the notion that COVID-19-induced MBC immunity is more robust than the vaccine-induced immunity, although clearly the latter is much safer and more predictable. Our data may therefore be of interest to the public health policy-making agencies when planning the schedule of COVID-19 vaccination.

## Figures and Tables

**Table 1:** Participant characteristics.

Study Group	SARS-CoV-2-Naïve, Gam-COVID-Vac (N/V)	SARS-CoV-2- Infected, Gam-COVID-Vac (PI/V)	SARS-CoV-2- Infected, Unvaccinated (PI/unV)
Number of participants	15	13	26
Age, years, median, (range)	60	42	51
	(24–70)	(23–69)	(21–64)
Female	11	5	10
Male	4	8	16
Days after first dose of vaccination, median (range)	179 (166–197)	175 (167–193)	-
Days after recovery median (range)	-	-	185 (178–208)
Days between infection and vaccination days, median (range)	-	102 (53–178)	-
PCR-confirmed COVID-19	-	13/13	26/26
Anti-Nucleocapsid antibodies before vaccination	0/15	13/13	-
Infection period	-	August 2020–February 2021	May 2020–January 2021
COVID-19 severity	-		
Mild		13	11
Moderate		0	13
Severe		0	2

## Data Availability

The data that support the findings of this study are available from the corresponding author upon reasonable request.

## Supplementary Materials

The following supporting information can be downloaded at: https://www.mdpi.com/article/10.3390/cells11131991/s1, Figure S1: Study design. Figure S2: Production of RBD-specific IgA (left panel) or lgM (right panel) in cultures of IL-21/CD40L-stimulated B cells evaluated using ELISA.
