# Supplementary material for: Functional Profiling of In Vitro Reactivated Memory B Cells Following Natural SARS-CoV-2 Infection and Gam-COVID-Vac Vaccination
Source: Cells. 2022 Jun 21;11(13):1991. doi: 10.3390/cells11131991 (PMC9265778; doi:10.3390/cells11131991)
Supplement: Supplementary file 1 [file cells-11-01991-s001.zip › cells-1705631-supplementary.pdf]

cells MDPI

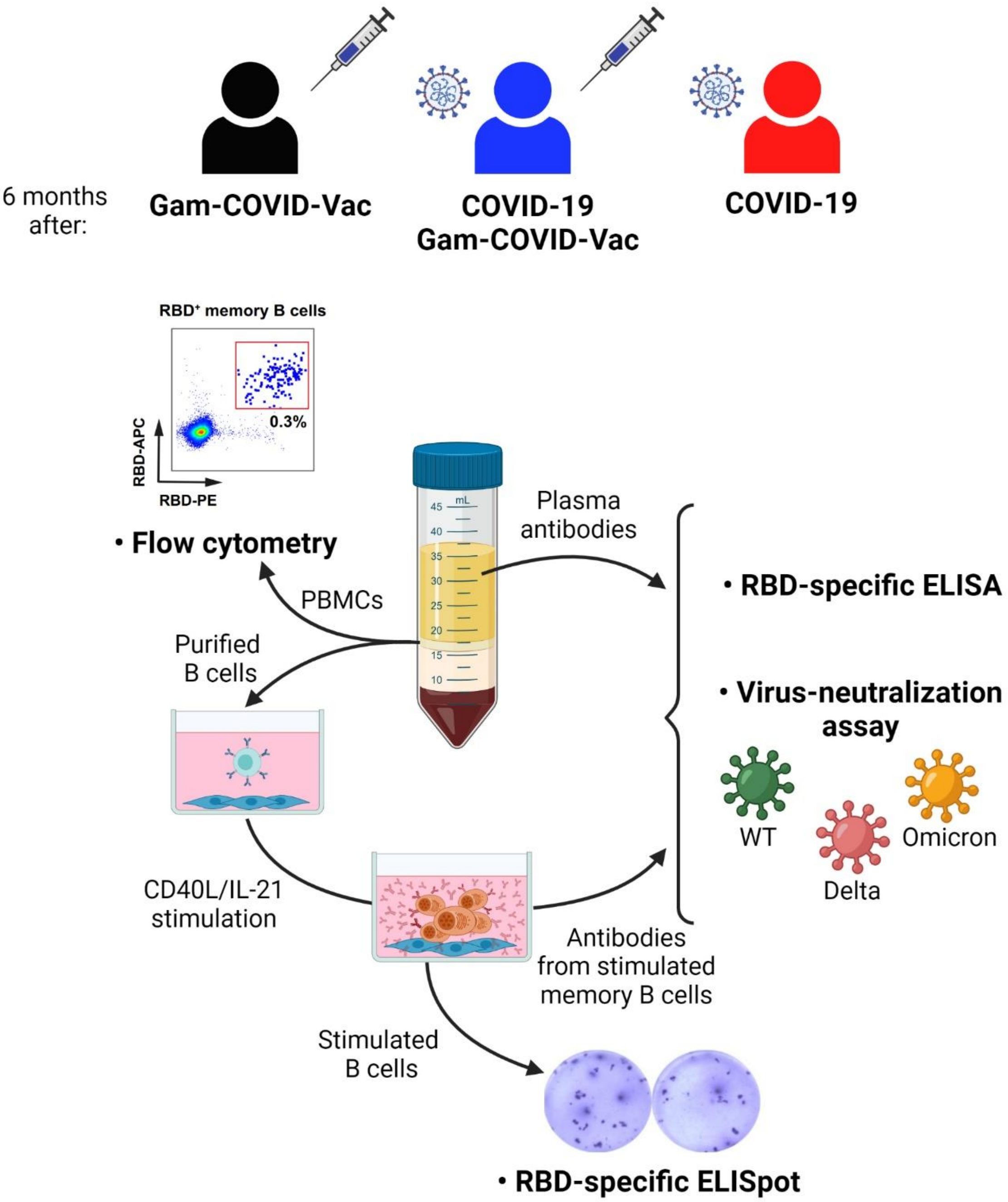

Figure S1. Study design.

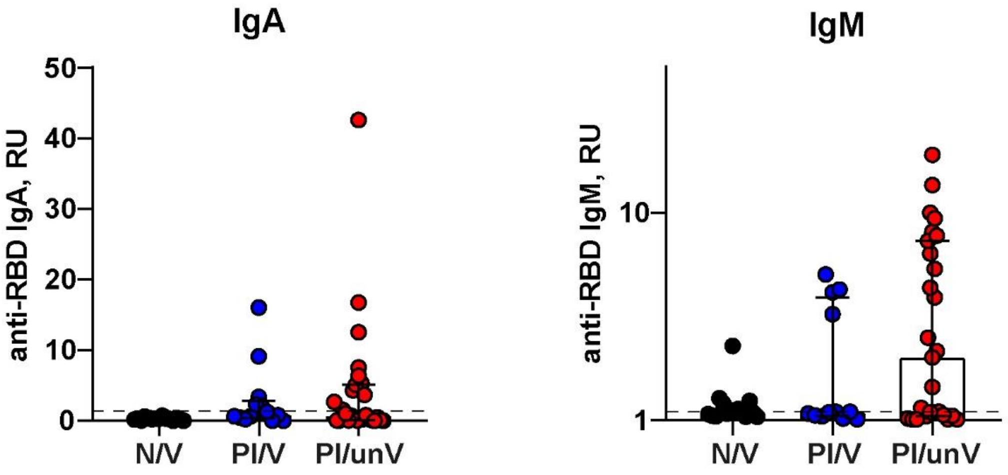

Figure S2. Production of RBD-specific IgA (left panel) or IgM (right panel) in cultures of IL-21/CD40L-stimulated B cells evaluated using ELISA.
